# Diel patterning in the bacterial community associated with the sea anemone *Nematostella vectensis*

**DOI:** 10.1002/ece3.5534

**Published:** 2019-08-13

**Authors:** Whitney B. Leach, Tyler J. Carrier, Adam M. Reitzel

**Affiliations:** ^1^ Department of Biological Sciences University of North Carolina at Charlotte Charlotte NC USA

**Keywords:** cnidarian, diel, immune, microbiome, photoperiod

## Abstract

Microbes can play an important role in the physiology of animals by providing essential nutrients, inducing immune pathways, and influencing the specific species that compose the microbiome through competitive or facilitatory interactions. The community of microbes associated with animals can be dynamic depending on the local environment, and factors that influence the composition of the microbiome are essential to our understanding of how microbes may influence the biology of their animal hosts. Regularly repeated changes in the environment, such as diel lighting, can result in two different organismal responses: a direct response to the presence and absence of exogenous light and endogenous rhythms resulting from a molecular circadian clock, both of which can influence the associated microbiota. Here, we report how diel lighting and a potential circadian clock impacts the diversity and relative abundance of bacteria in the model cnidarian *Nematostella vectensis* using an amplicon‐based sequencing approach. Comparisons of bacterial communities associated with anemones cultured in constant darkness and in light:dark conditions revealed that individuals entrained in the dark had a more diverse microbiota. Overall community composition showed little variation over a 24‐hr period in either treatment; however, abundances of individual bacterial OTUs showed significant cycling in each treatment. A comparative analysis of genes involved in the innate immune system of cnidarians showed differential expression between lighting conditions in *N. vectensis,* with significant up‐regulation during long‐term darkness for a subset of genes. Together, our studies support a hypothesis that the bacterial community associated with this species is relatively stable under diel light conditions when compared with static conditions and that particular bacterial members may have time‐dependent abundance that coincides with the diel photoperiod in an otherwise stable community.

## 1 INTRODUCTION

Animals and other eukaryotes associate with diverse microbial communities that are known to have distinct and sometimes essential roles in the development, physiology, and life history of various species (Fraune & Bosch, 2010; Kohl & Dearing, 2012; Macke, Tasiemski, Massol, Callens, & Decaestecker, 2017; McFall‐Ngai & Ruby, 2000; Sommer & Backhed, 2013). The members that compose host‐associated microbial communities often shift depending on the local environmental conditions (Carrier & Reitzel, 2017), the presence of particular species that may facilitate or limit the colonization by other microbes (Vega & Gore, 2017), and the expression of the immune system by the host (Nyholm & Graf, 2012; Thaiss, Zmora, Levy, & Elinav, 2016). Over the past few decades, sequence‐based approaches have broadened our understanding of diverse interactions between hosts and associated microbial communities (O'Brien, Webster, Miller, & Bourne, 2019). Specifically, these studies have provided insight into the relative proportions of microbes that are stably symbiotic or transient with a host when experiencing variable environmental conditions (Shade & Handelsman, 2012), including external factors (e.g., temperature, nutrients) or host‐regulation (e.g., immune system). Determining how these factors impact host‐associated microbial communities in general, and how they affect specific OTUs (operational taxonomic units), would provide a better understanding of how complex microbial communities vary for eukaryotes.

Light is an environmental factor that influences many organisms through a combination of two principal responses. First, light can significantly impact the physiology and survival of an organism following direct exposure, and photosynthetically active wavelengths may impact the function of microbial partners. The result can be positive for increasing growth of certain microbes (e.g., cyanobacteria), where photons are harvested for the production of photosynthates. Light can also have negative effects by causing damage that can inhibit growth, particularly for short wavelength portions of the light spectrum (Dai et al., 2012). Secondly, animal‐associated bacterial communities can, in turn, shift following responses by the host due to an entrained endogenous pathway (the circadian clock). Circadian rhythms are critical internal regulatory systems that allow organisms to anticipate daily changes in their environment and adjust biological processes appropriately. Using an endogenous centralized clock, cycles of about 24‐hr are entrained and maintained by exogenous cues (Zeitgebers) that modulate temporal rhythms through a series of transcription–translation feedback loops (Dunlap, 1999). Previous work with vertebrates suggests that the circadian clock is an important regulator of the immune system, which can impact portions of the bacterial community throughout a day. In humans (Huang, Ramsey, Marcheva, & Bass, 2011) and mice (Leone et al., 2015), the gut microbiota is time‐of‐day–dependent and is hypothesized to modulate the regulation of host metabolism and immunity (Keller et al., 2009; Liang, Bushman, & FitzGerald, 2015; Thaiss et al., 2014). However, the interaction between light‐dependent responses that influence the host's behavior and an endogenous circadian clock remains unknown.

Our knowledge of the connections between diel lighting, circadian rhythms, and symbiotic microbiota remains limited in invertebrates, and especially those in aquatic habitats. One of the best‐studied examples is the mutualism between the squid *Euprymna scolopes* and bacterium *Aliivibrio fischeri* (formally *Vibrio fischeri*; Boettcher, Ruby, & McFall‐Ngai, 1996; Heath‐Heckman et al., 2013; Wier et al., 2010), where the expression of a light‐sensitive cryptochrome (*escry1*) in the host has been linked to the presence of *A. fischeri*. While the *Euprymna‐Aliivibrio* system provides a host‐focused view of circadian‐related symbioses, decades of work on the coral microbiome have provided additional context for the evolutionary ecology of daily cycles exhibited by both the holobiont and by each partner (Hoadley, Vize, & Pyott, 2016). *Symbiodiniaceae*, a eukaryotic endosymbiotic mutualist of corals and other invertebrates, demonstrates diel periodicity of photosynthetic processes in the free‐living and mutualistic states, suggesting that symbiotic partners maintain their own circadian clocks and, perhaps, contribute to that of the holobiont (Roopin, Yacobi, & Levy, 2013; Sorek, Díaz‐Almeyda, Medina, & Levy, 2014; Sorek, Yacobi, Roopin, Berman‐Frank, & Levy, 2013). Aside from these systems, oscillations of individual members or communities of host‐associated microbiota in marine invertebrates are poorly understood. Further, how much of the holobiont rhythmicity is due to a direct response to environmental cues (e.g., light) or is driven by endogenous mechanisms remains an active area of research (Brady, Willis, Harder, & Vize, 2016; Leach, Macrander, Peres, & Reitzel, 2018; Oren et al., 2015; Vize, 2009).

*Nematostella vectensis*, an infaunal sea anemone that lives in shallow estuaries, is an emerging model for studying the host‐associated microbial communities and circadian biology of cnidarians. Similar to corals, *N. vectensis* exhibits nocturnal patterns in behavior [e.g., circadian locomotion and body expansion (Hendricks, Byrum, & Meyer‐Bernstein, 2012; Oren et al., 2015)], gene expression [e.g., immunity and stress tolerance (Leach et al., 2018)], and metabolism (Maas, Jones, Reitzel, & Tarrant, 2016). Unlike corals, *N. vectensis* does not associate with zooxanthellate and, thus, exhibits rhythmicity independent of the eukaryotic mutualist of corals and other cnidarians. Previous studies have shown that the bacterial community associated with *N. vectensis* is diverse in natural habitats (Har et al., 2015), variable across development (Mortzfeld et al., 2016), and significantly dissimilar for individuals from different geographic locations (Mortzfeld et al., 2016), which together support this species as a system to study animal and bacterial interactions (Fraune, Foret, & Reitzel, 2016).

The innate immune system is a combination of molecular mechanisms that may explain the variation in bacteria associated with cnidarians (Bosch et al., 2009). Genomic and transcriptomic resources for a number of anthozoan and hydrozoan species have identified numerous genes predicted to be involved in cnidarian immunity (Miller et al., 2007; Reitzel, Sullivan, Traylor‐Knowles, & Finnerty, 2008). Based on sequence similarity and experimental characterization, cnidarians have many components of a traditionally defined innate immune system, including the Toll‐like and NOD‐like receptors for microbial recognition (Bosch et al., 2009; Brennan et al., 2017), at least three complement families [e.g., C3, Bf, and MASP (Kimura, Sakaguchi, & Nonaka, 2009), MyD88 and other proteins for intracellular signal transduction (Franzenburg et al., 2012), and Nf‐κB along with other Rel‐related proteins for transcriptional regulation of effector genes (Sullivan et al., 2009; Wolenski et al., 2011)]. These studies support the hypothesis that the cnidarian–bilaterian ancestor had a rich and complex innate immune system. The regulation of the cnidarian system and how environmental changes may modulate the expression of components of this pathway remain unstudied.

Using high‐throughput sequencing of the 16S rDNA gene to represent the bacterial communities associated with *N. vectensis*, we tested two hypotheses regarding if and how diel lighting influences the anemone‐associated bacterial community. First, we tested whether the bacterial community of *N. vectensis* exhibits diel oscillations synchronous with light:dark cycling, and second, whether individual OTUs were differentially abundant after host exposure to light:dark cycles. Here, we identify compositional differences between anemones exposed to light:dark cycles or constant darkness. Further, these data reveal specific bacterial OTUs that exhibit diel patterns of abundance in either light regime. By assessing bacterial abundance across diel and constant conditions, our research sheds light on the potential of microbial interactions in the regulation of host anemone cyclic behavior and physiology measured in other studies (Hendricks et al., 2012; Maas et al., 2016; Oren et al., 2015).

## 2 MATERIALS AND METHODS

### 2.1 Animal culturing and experimental conditions

Adult *Nematostella vectensis* derived from the original “Maryland strain” (Putnam et al., 2007) were used for these experiments. Individuals were reared in a single dish at room temperature (~20–25°C) and ambient lighting conditions (as described in Hand & Uhlinger, 1994). In preparation for the experiment, adults from the common garden conditions were split into two glass dishes and transferred to an incubator at 25°C. Individuals were fed freshly hatched brine shrimp (*Artemia *sp.) haphazardly three times weekly, and water was replaced following feeding using 200 ml of 15 ppt artificial saltwater.

**Figure 1:**
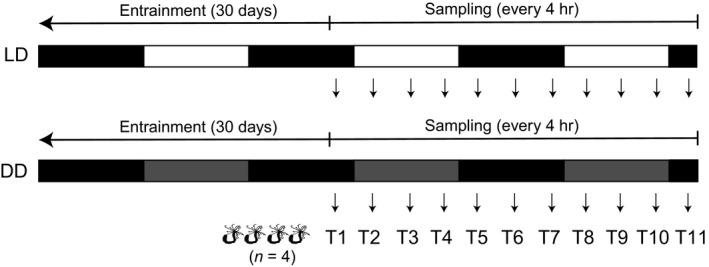
Experimental design for light:dark (LD) and constant dark conditions (DD) and corresponding sampling

To simulate diel light conditions, full‐spectrum LED lights (MINGER) were set to 12‐hr light:12‐hr dark (LD) cycles, with lights on at 11:30 a.m. (ZT = 0) and lights off (ZT = 12) at 11:30 p.m. Each dish of individuals was assigned to LD or DD (constant darkness) and was subsequently kept in their respective conditions for 30 days (Figure 1). During this entrainment period, individuals were kept on the same feeding and water change schedule as previous. Feeding and water changes occurred during “daytime” hours (between ZT = 0 and ZT = 12). To eliminate the potential for light contamination in DD animals, dishes were wrapped in tin foil during the entrainment and sampling periods; however, dishes were briefly removed from the incubator and were handled in a dark room for feeding and water changes.

Two days prior to sampling, individuals from each light regime were split into four glass dishes per condition (LD 1‐4; DD 1‐4) with 200 ml of fresh, 15 ppt artificial seawater and were starved. Beginning at 9:30 a.m. (ZT = 22), individuals from each dish were sampled at four‐hour intervals for a total of 11 time points (Figure 1). Four biological replicates per time point (A‐D, one per bowl) per condition (LD and DD) were collected for a total 88 samples. Samples were preserved in RNAlater at −20°C until processing.

### 2.2 Assaying microbial communities

Total DNA was extracted from *N. vectensis* samples using the Qiagen All Prep Kit (Thermo Scientific), quantified using the NanoDrop 2000 UV‐Vis Spectrophotometer (Thermo Scientific), and standardized to 5 ng/μl using RNase/DNase‐free water.

Bacterial sequences were amplified using universal primers for the V3/V4 regions of the 16S rDNA gene (Forward: 5′ CCTACGGGNGGCWGCAG, Reverse: 5′ GACTACHVGGGTATCTAATCC (Klindworth et al., 2013; see Dryad for Table S1). Products were purified using the Axygen AxyPrep Mag PCR Clean‐up Kit (Axygen Scientific), indexed via PCR using the Nextera XT Index Kit V2 (Illumina Inc.), and then purified again. At each of these three cleanup steps, fluorometric quantification was performed using a Qubit (Life Technologies), and libraries were validated using a Bioanalyzer High Sensitivity DNA chip (Agilent Technologies). Illumina MiSeq sequencing (v3, 2x300 bp paired‐end reads) was performed at the University of North Carolina at Charlotte.

Forward and reverse sequences were paired and trimmed using PEAR (Zhang, Kobert, Flouri, & Stamatakis, 2014) and Trimmomatic (Bolger, Lohse, & Usadel, 2014), respectively, converted from fastq to fasta using a custom script (see Dryad for Supplemental Note), and, prior to analysis of bacterial 16S rDNA sequences, chimeric sequences were detected using USEARCH (Edgar, Haas, Clemente, Quince, & Knight, 2011) and removed using filter_fasta.py. Using QIIME 1.9.1 (Caporaso et al., 2010) and SILVA (Quast et al., 2013), bacterial 16S rDNA sequences were grouped into operational taxonomic units (OTUs) based on a minimum 99% similarity. The biom table generated by pick_open_reference_otus.py was filtered of OTUs with less than ten reads, as well as “unassigned” sequences.

Using the filtered biom table and “biom summarize‐table” function to count total sequences per sample, the rarefaction depth of 1,080 (see Dryad for Figure S1) was determined and applied to all subsequent analyses. Alpha diversity (i.e., McIntosh dominance index, McIntosh evenness index, Menhinick richness index, Faith's phylogenetic distance, and observed OTUs) was calculated using alpha_diversity.py and compared statistically using Student's *t* test in JMP. Beta diversity was calculated using unweighted and weighted UniFrac (Lozupone & Knight, 2005), compared using principal coordinate analyses (PCoA) with jackknifed_beta_diversity.py, visualized using make_2d_plots.py, and stylized for presentation in Adobe Illustrator CS6. UniFrac distances were then compared statistically using an analysis of similarity (ANOSIM) in QIIME as part of compare_categories.py. Community composition was generated using summarize_taxa_through_plots.py and stylized using Prism 7 (GraphPad Software) and Adobe Illustrator CS6. Differential abundance of OTUs between light regimes was tested using the DESeq2_nbinom algorithm as part of differential_abundance.py. Lastly, the shared or “core” community was determined using compute_core_microbiome.py and shared_phylotypes.py. Venn diagrams showing shared and unique OTUs were generated by comparing taxa between treatments.

A step‐by‐step listing of the informatic pipeline, including QIIME scripts, used to convert and process raw reads are available on Dryad in file “Supplemental Note.”

### 2.3 Identification of oscillating OTUs

Using the filtered biom table, we identified oscillating OTUs using the R statistical package JTK_Cycle (version 3.1; Hughes, Hogenesch, & Kornacker, 2010). Specifically, we used the script described by Hughes et al. (2010), setting the parameters to select significantly cycling OTUs between 20 and 28 hr (JTK_Cycle, *p* < .05; “per” = 24) and shifts in peak expression (JTK_Cycle, “lag”) between LD and DD samples were compared (Zeitgeber Time: ZT). JTK_Cycle does not classify units into rhythmic categories; therefore, we compared read counts for OTUs based on their periodicity values (“per”) and significance (*p*‐value). ANOVA and post hoc Tukey tests were performed with GraphPad Prism between timepoints and within treatments.

## 3 RESULTS

To characterize the variation in the bacterial community associated with *N. vectensis* when cultured in two treatments, light:dark (LD) and constant dark (DD), we used 16S rDNA sequencing to compare microbial diversity and abundance in each light regime.

### 3.1 Community‐level dynamics

**Figure 2:**
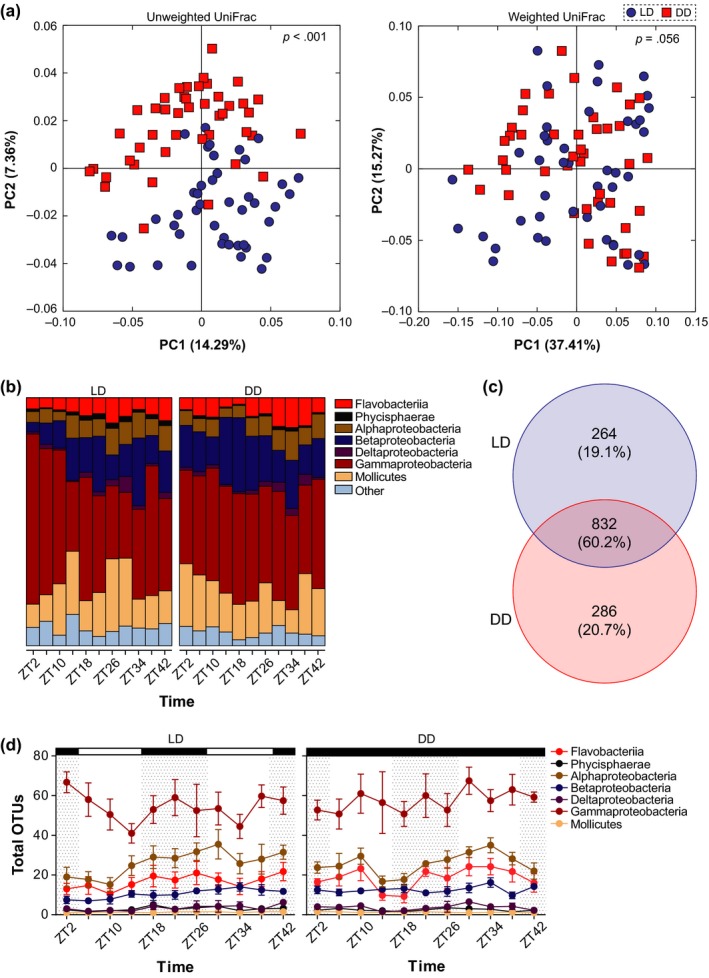
Similarity amongst the bacterial community associated with light:dark (LD) and constant darkness (DD) entrained *Nematostella vectensis*. (a) Community similarity for *N. vectensis* entrained to a diel photoperiod and constant conditions based on unweighted and weighted UniFrac metric. (b) Bacterial classes associated with *N. vectensis* when entrained to LD or DD that represent at least 1% of the community (with classes representing less than 1% of the community grouped under “other”). (c) Bacterial OTUs that were either LD‐specific, DD‐specific, or shared between the two conditions. (d) Total OTUs for each bacterial class (that represents at least 1% of the community)

Differences in the bacterial communities associated with *N. vectensis* were best explained by the presence of a diel photoperiod (Figure 2a; ANOSIM, unweighted UniFrac: *p* = 0.001; ANOSIM, weighted UniFrac: *p* = 0.056). When comparing alpha diversity between LD and DD, communities were similar in dominance (*t* test, McIntosh dominance index: *p* = 0.580), evenness (*t* test, McIntosh evenness index: *p* = 0.520), richness (*t* test, Menhinick richness index: *p* = 0.297), and observed OTUs (*t* test; *p* = 0.078) (see Dryad for Table S2). Anemones cultured in constant darkness, however, associated with a bacterial community that was 13% more phylogenetically diverse than anemones cultured under light:dark conditions (*t* test, Faith's phylogenetic distance: *p* = 0.047; see Dryad for Table S2).

There was no difference in the bacterial taxa (membership; ANOSIM, unweighted UniFrac: *p* = 0.418) or their relative abundance (composition; ANOSIM, weighted UniFrac: *p* = 0.798) between day and night periods in LD. However, there was dissimilarity in membership (ANOSIM, unweighted UniFrac: *p* = 0.046), but not composition (ANOSIM, weighted UniFrac: *p* = 0.329) for DD individuals. Moreover, when comparing bacterial communities across all sampled time points, we observed no differences in LD membership or composition (ANOSIM, unweighted UniFrac: *p* = 0.280; ANOSIM, weighted UniFrac: *p* = 0.309), but time‐dependent differences were observed in membership for DD entrained individuals (ANOSIM, unweighted UniFrac: *p* = 0.0016; ANOSIM, weighted UniFrac: *p* = 0.329).

Lastly, the community‐level pattern observed for LD or DD was not confounded by differences between biological replicates ("bowl effects"). Specifically, there was no significant variation in the taxonomy and composition of *N. vectensis*‐associated bacterial communities under LD (ANOSIM, unweighted UniFrac: *p* = 0.082; ANOSIM, weighted UniFrac: *p* = 0.170) and DD (ANOSIM, unweighted UniFrac: *p* = 0.892; ANOSIM, weighted UniFrac: *p* = 0.925) conditions.

### 3.2 Composition of the bacterial community

The bacterial communities associated with *N. vectensis* were best explained by the presence/absence of a diel photoperiod, so we next compared the taxonomic profiles of bacteria in each lighting treatment. The bacterial communities of anemones in LD consisted primarily of seven bacterial classes: Gammaproteobacteria (Proteobacteria; 55.70%), Mollicutes (Tenericutes; 13.40%), Betaproteobacteria (Proteobacteria; 10.70%), Flavobacteriia (Bacteroidetes; 5.90%), Alphaproteobacteria (Proteobacteria; 5.70%), Deltaproteobacteria (Proteobacteria; 1.80%), and Phycisphaerae (Planctomycetes; 1.20%) (Figure 2b). The bacterial communities of anemones in DD primarily associated with the same seven bacterial classes but at different relative proportions: Gammaproteobacteria (Proteobacteria; 49.20%), Betaproteobacteria (Proteobacteria; 13.30%), Mollicutes (Tenericutes; 13.10%), Flavobacteriia (Bacteroidetes; 8.70%), Alphaproteobacteria (Proteobacteria; 6.80%), Deltaproteobacteria (Proteobacteria; 2.30%), and Phycisphaerae (Planctomycetes; 1.00%) (Figure 2b). Although we did observe treatment‐specific OTUs (Figure 2c), total OTUs within these abundant bacterial classes varied little between LD and DD as well as across time (Figure 2d; see Dryad for Table S3).

Differences in the bacterial communities of LD and DD entrained anemones were due, in part, to the differential abundance of 37 bacterial OTUs (Table 1). Of those 37 differentially abundant OTUs, 17 (45.9%) were overabundant in LD, while 20 (54.1%) were underabundant (Table 1). Relative to DD, the bacterial classes with overabundant OTUs that were in significantly higher proportions in LD included: Saprospirae and Flavobacteriia (Bacteroidetes), Chlamydiia (Chlamydiae), Lentisphaeria (Lentisphaerae), OM190 (Planctomycetes), and Alphaproteobacteria and Gammaproteobacteria (Proteobacteria) (Table 1). The bacterial classes with underabundant OTUs included the following: Flavobacteriia, Phycisphaerae and Planctomycetia (Planctomycetes), Alphaproteobacteria and Gammaproteobacteria, and Opitutae and Verrucomicrobiae (Verrucomicrobia) (Table 1).

**Table 1:** Taxonomic classification for differentially abundant OTUs associated with *Nematostella vectensis* in light:dark (LD) relative to constant darkness (DD) conditions

Phylum	Class	Order	Family	Genus	Log2 fold change	*p*‐value	Adjusted *p*‐value
**Overabundant**							
Bacteroidetes	Saprospirae	Saprospirales	Chitinophagaceae	–	0.813	2.30E‐06	3.30E‐05
	Flavobacteriia	Flavobacteriales	Flavobacteriaceae	*Aequorivita*	0.442	8.20E‐03	5.40E‐02
				*Flavobacterium*	0.73	3.40E‐05	4.20E‐04
				*Polaribacter*	0.959	7.50E‐08	1.60E‐06
				*Tenacibaculum*	0.658	7.40E‐05	8.50E‐04
Chlamydiae	Chlamydiia	Chlamydiales	–	–	1.502	1.90E‐18	1.60E‐16
Lentisphaerae	Lentisphaeria	Lentisphaerales	Lentisphaeraceae	–	0.471	7.30E‐03	5.20E‐02
Planctomycetes	OM190	CL500‐15	–	–	0.611	4.10E‐04	3.90E‐03
Proteobacteria	Alphaproteobacteria	Kiloniellales	–	–	0.429	1.30E‐02	7.10E‐02
			Kiloniellaceae	–	0.852	2.90E‐07	5.00E‐06
	Gammaproteobacteria	Alteromonadales	Alteromonadaceae	*Alteromonas*	0.445	1.20E‐02	6.70E‐02
			Colwelliaceae	*Thalassomonas*	1.239	2.70E‐13	1.60E‐11
			Shewanellaceae	*Shewanella*	1.229	6.70E‐12	2.90E‐10
					0.467	6.50E‐03	4.80E‐02
					0.411	1.60E‐02	8.20E‐02
		Oceanospirillales	Oceanospirillaceae	*Oleibacter*	0.467	8.20E‐03	5.40E‐02
		Pseudomonadales	Pseudomonadaceae	*Pseudomonas*	1.024	8.80E‐09	2.20E‐07
**Underabundant**							
Bacteroidetes	Flavobacteriia	Flavobacteriales	Cryomorphaceae	*Fluviicola*	−0.416	1.80E‐02	8.50E‐02
			Flavobacteriaceae	–	−0.44	1.00E‐02	6.20E‐02
				*Polaribacter*	−0.849	1.00E‐06	1.60E‐05
				*Sediminicola*	−0.515	1.90E‐03	1.60E‐02
			–	–	−0.937	8.30E‐08	1.60E‐06
Cyanobacteria^a^	Chloroplast	Stramenopiles	–	–	−1.71	1.30E‐21	2.20E‐19
			–	–	−1.085	7.70E‐10	2.20E‐08
Planctomycetes	Phycisphaerae	Phycisphaerales	Phycisphaeraceae	–	−0.599	1.90E‐04	2.00E‐03
				–	−0.512	1.60E‐03	1.50E‐02
	Planctomycetia	Pirellulales	Pirellulaceae	–	−0.44	1.20E‐02	6.90E‐02
Proteobacteria	Alphaproteobacteria	Rhizobiales	Bradyrhizobiaceae	–	−0.418	1.50E‐02	8.00E‐02
		Rhodobacterales	Rhodobacteraceae	–	−0.408	1.70E‐02	8.30E‐02
		Sphingomonadales	–	–	−0.479	5.30E‐03	4.10E‐02
	Gammaproteobacteria	Alteromonadales	Alteromonadaceae	–	−0.811	4.90E‐06	6.50E‐05
		Oceanospirillales	Oceanospirillaceae	*Neptunomonas*	−0.538	2.40E‐03	2.00E‐02
		Vibrionales	Pseudoalteromonadaceae	–	−0.447	9.20E‐03	5.80E‐02
			Vibrionaceae	*Photobacterium*	−0.422	1.80E‐02	8.50E‐02
				*Vibrio*	−0.464	9.50E‐03	5.80E‐02
Verrucomicrobia	Opitutae	Puniceicoccales	Puniceicoccaceae	*Coraliomargarita*	−0.648	2.50E‐04	2.50E‐03
	Verrucomicrobiae	Verrucomicrobiales	Verrucomicrobiaceae	*Persicirhabdus*	−1.1	4.10E‐10	1.40E‐08

a Potential algal contaminant.

### 3.3 Shared taxa between LD and DD

A shared (or “core”) bacterial community for *N. vectensis* in LD and DD was determined for different proportions of shared OTUs. At a core level of 60%, 70%, 80%, 90%, and 100% (i.e., bacterial phylotypes found in at least “*N*”% of samples), we observed that 141, 93, 63, 38, and nine phylotypes (see Dryad for Figure S2), respectively, were shared between LD and DD conditions. At core levels 60% and 70%, we observed that the taxonomic representation (but not composition) of these communities was distinct but converged at a core level of 80% (see Dryad for Figure S3).

The taxonomic composition of the “core” community was dominated by three bacterial classes that were also common in the full communities: Gammaproteobacteria (Proteobacteria), Mollicutes (Tenericutes), and Betaproteobacteria (Proteobacteria) (see Dryad for Figure S4). In these core communities, we also observed Actinobacteria (Actinobacteria), Flavobacteriia (Bacteroidetes), Lentisphaeria (Lentisphaerae), OM190 and Phycisphaerae (Planctomycetes), Alphaproteobacteria and Deltaproteobacteria (Proteobacteria), Spirochaetes (Spirochaetes), and Opitutae (Verrucomicrobia) (see Dryad for Figure S4).

### 3.4 Patterns of abundance in LD and DD

**Figure 3:**
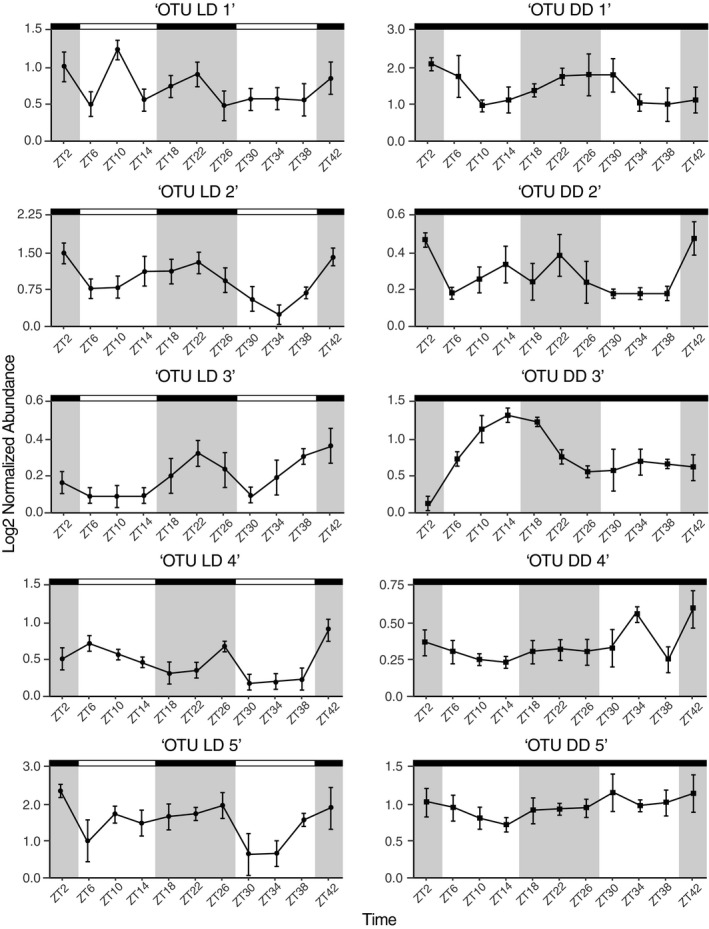
Abundance plots of 24‐hr cycling bacterial OTUs during light:dark (left panel; black and white boxes) and constant darkness (right panel; solid black boxes) over the time course. Gray shaded areas indicate night and white areas indicate day. OTU names have been simplified and full names can be found in Table 2

Using JTK_cycle (Hughes et al., 2010), we identified 26 bacterial OTUs in LD that showed rhythmic cycling (*p* < 0.05), five of which exhibited a 24‐hr periodicity with peak abundance at either ZT = 20 or ZT = 22 (per = 24; Figure 3; Table 2). Of these five OTUs, four were from the bacterial order Rhodobacterales and the other was from Alteromonadales (Table 2). In DD, 16 bacterial OTUs showed rhythmic cycling (*p* < 0.05), and five of these exhibited 24‐hr periodicity with peak abundance ranging between ZT = 2 and ZT = 20 (per = 24; Figure 3; Table 2). Unlike the cycling OTUs in LD, OTUs in DD with 24‐hr cycling were from four disparate bacterial orders: Chlamydiales, Spirochaetales, Oceanospirillales, and CL500‐15 (Table 2). When comparing the ten bacterial OTUs that exhibited a 24‐hr periodicity to the core community, we observed that OTUs “LD 1,” “LD 2,” and “LD 5” were specific to LD, while OTU “DD 2” was specific to DD. Moreover, OTUs “LD 2” and “LD 5” were observed at the 80% core level, while the OTU “DD 2” was only detected at the 60% core level.

**Table 2:** Taxonomy of OTUs with significant 24‐hr periodicity associated with adult *Nematostella vectensis* in light:dark (LD) and constant darkness (DD) conditions, as determined by JTK_Cycle

	**Operational Taxonomic Unit**	**Phylum**	**Class**	**Order**	**Family**	**Genus**
**LD**						
OTU LD 1	New.ReferenceOTU9413	Proteobacteria	Alphaproteobacteria	Rhodobacterales	Rhodobacteraceae	–
OTU LD 2	DQ234245.1.1495	Proteobacteria	Alphaproteobacteria	Rhodobacterales	Rhodobacteraceae	–
OTU LD 3	DQ234200.1.1520	Proteobacteria	Alphaproteobacteria	Rhodobacterales	Rhodobacteraceae	–
OTU LD 4	KX874549.1.1479	Proteobacteria	Alphaproteobacteria	Rhodobacterales	Rhodobacteraceae	–
OTU LD 5	CP010912.3828314.3829852	Proteobacteria	Gammaproteobacteria	Alteromonadales	Alteromonadaceae	*Alteromonas*
**DD**						
OTU DD 1	New.ReferenceOTU22615	Chlamydiae	Chlamydiia	Chlamydiales	–	–
OTU DD 2	JN713484.1.1490	Spirochaetes	Spirochaetes	Spirochaetales	Spirochaetaceae	–
OTU DD 3	JQ999980.1.1535	Proteobacteria	Gammaproteobacteria	Oceanospirillales	Alcanivoracaceae	*Alcanivorax*
OTU DD 4	FPLL01004330.9.1533	Chlamydiae	Chlamydiia	Chlamydiales	–	–
OTU DD 5	New.ReferenceOTU16126	Planctomycetes	OM190	CL500‐15	–	–

## 4 DISCUSSION

Photoperiods and circadian clocks are an integral part of diverse biological processes for animals, ranging from immune performance to metabolism to host–microbe associations (Heath‐Heckman, 2016; Hubbard et al., 2018; Liang et al., 2015; Zarrinpar, Chaix, Yooseph, & Panda, 2014). In traditional mammalian systems, such as mice and humans, the composition of the gut microbiota of individuals entrained to light:dark or constant darkness differs for particular taxonomic groups of bacteria (Deaver, Eum, & Toborek, 2018; Wu et al., 2018). Thus, the photoperiod may influence compositional dynamics of host‐associated bacterial communities in other animals, and the responses may also involve an endogenous circadian clock. While *N. vectensis*, like other cnidarians, has well described rhythmic behavior, physiology, and gene expression under light entrainment (Hendricks et al., 2012; Maas et al., 2016; Oren et al., 2015; Reitzel, Behrendt, & Tarrant, 2010), this is the first investigation of potential rhythmicity in their associated bacterial community. Here, we show that *N. vectensis* entrained to constant darkness associated with a phylogenetically more diverse bacterial community than anemones entrained to light:dark. Moreover, we find support that the relative abundance of a limited number of OTUs oscillated over the course of a day (Figure 3). Of those OTUs specific to light:dark conditions, four were within the bacterial order Rhodobacterales, while those oscillating in constant conditions were phylogenetically disparate (Table 2).

The research we report here suggests that diel lighting may impact a fraction of the microbial community, but this effect appears to be relatively small compared with differences in the associated microbiota over developmental stages or in natural populations (Har et al., 2015; Mortzfeld et al., 2016). For *N. vectensis* and other animals, the role of the animal host, the environment, and the resident microbiota may play in shaping host–microbe interactions remains fragmentary. One hypothesis for the observed shift in community‐level composition reported here is that the lack of a photoperiod drives ecological (or stochastic) drift in the microbes associated with *N. vectensis*. Generally, in ecological systems, species diversity is expected to increase as environmental heterogeneity increases up to a point as described in Curd, Martiny, Li, and Smith (2018). Our results do not show a positive relationship between environmental variation (i.e., diel lighting) and community diversity; rather, we measured greater community‐level variability in constant conditions (Table 2).

A second hypothesis for community‐level shifts over a diel light period is that changes in bacteria are attributed to physiological differences between individuals in light:dark and constant darkness (i.e., gene expression, behavior, metabolism; Leach et al., 2018; Maas et al., 2016; Peres et al., 2014; Reitzel et al., 2010; Roopin & Levy, 2012). The photoperiod may influence compositional dynamics of host‐associated bacterial communities through differential regulation of the immune system, potentially through an endogenous circadian clock. Studies in *Hydra* have shown that immune factors and bacteria–bacteria interactions are critical for function in restricting membership of the microbiome (Augustin et al., 2017; Bosch et al., 2009; Franzenburg et al., 2012; Fraune et al., 2015). While previous research has shown that *N. vectensis* has a circadian clock based on behavioral, physiology, and molecular measurements (see Section 1), genes likely to be involved in innate immunity have little differential expression over a 24‐hr period, at least when measured in whole animal homogenates. As a preliminary investigation using previously published transcriptomic data (Leach & Reitzel, 2019), we compared the expression of candidate cnidarian immune genes from anemones sampled in LD and DD conditions. The genes selected include the hypothesized principal innate immune genes (e.g., Toll‐like receptors, Nf‐κB) from Miller et al. (2007) and Brennan et al. (2017), the NOD‐like receptors from Lange et al. (2011) and Yuen, Bayes, and Degnan (2014), and the complement genes identified by Kimura et al. (2009) (Table 3). These transcriptomic comparisons showed only a small portion of the genes predicted to be involved in the cnidarian innate immune system (7 out of the 34 surveyed) to be differently expressed between LD and DD (Table 3). Of these seven, four genes were up‐regulated in constant dark conditions (compared to LD) and included predicted members of the cnidarian multi‐complement pathway (e.g., *NvC3‐1*, *NvBF1*, and *NvBF2*) and NOD‐like receptors. The function of any of these genes in *N. vectensis* is unknown, but the complement genes have spatially restrictive expression in the endoderm (Kimura et al., 2009). Overall, similarity in the expression of immune genes may explain the consistency of the microbial community between lighting treatments. The small number of OTUs showing 24‐hr oscillating abundance could be a result of the subset of differentially expressed immune genes, which could be tissue specific.

**Table 3:** Differential gene expression of candidate cnidarian innate immune genes between light:dark (LD) and constant darkness (DD) determined by DESeq2. NOD genes were arbitrarily given numbers and the order matches the order in Table S1 from Lange et al. (2011)

Annotation	JGI	log2 fold change^a^	*p*‐value^b^
**BF1**	**41116**	**−0.7869**	**0.0084**
**BF2**	**204186**	**−0.3549**	**0.0393**
**C3‐1**	**18**	**−1.1465**	**0.0002**
IkB	–	NA	NA
IKK	163386	0.0700	0.4012
LBP	170435	−0.0743	0.5176
MASP	138799	NA	NA
Myd88	82163	0.1660	0.1944
NFkB	174238	NA	NA
**RIGIa**	**87071**	**0.3639**	**0.0051**
TAB2	233007	0.1264	0.1200
TAK1 (MAP3K7)	87118	−0.0534	0.7785
TLR1	16780	NA	NA
TLR2	201237	NA	NA
TLR3	196737	NA	NA
TLR4	204009	NA	NA
TRAF6	178259	0.1389	0.3813
Nod_2	160179	−0.1109	0.4910
**Nod_23**	**220102**	**−0.4177**	**0.0077**
Nod_29	248679	0.0308	0.9093
Nod_30	242728	0.0705	0.7583
**Nod_31**	**247590**	**0.2500**	**0.0210**
Nod_35	240600	0.4993	0.1402
**Nod_36**	**240601**	**0.2482**	**0.0486**
Nod_51	94204	0.0571	0.7559
Nod_53	87696	−0.1262	0.3731
Nod_54	60625	0.1746	0.2458
Nod_55	138346	0.0657	0.7723
Nod_61	247717	0.1617	0.1242
Nod_62	215101	0.1173	0.2788
Nod_63	246451	−0.1069	0.4213
Nod_66	244932	−0.0894	0.4993
Nod_68	239890	0.1620	0.2123

Note

NA genes did not meet the mean count cutoff for DESeq2.

Bold text indicates significant up‐ or down‐regulation.

a Pairwise comparisons using Wald tests in DESeq2.

b Benjamini–Hochberg FDR corrected *p*‐value.

Complementary to photoperiod‐related shifts in cnidarian‐associated microbiota we report here, a number of studies have shown that additional rhythmic abiotic factors may affect compositional changes in these symbiont communities (Cai et al., 2018; Sharp, Pratte, Kerwin, Rotjan, & Stewart, 2017; Silveira et al., 2017; Sweet, Brown, Dunne, Singleton, & Bulling, 2017). At present, we have a rudimentary understanding of what drives the observed specificity between a host and their associated species‐specific microbial communities. When comparing the bacterial communities of *N. vectensis* across development, environmental conditions, and geographic locations, Mortzfeld et al. (2016) and Domin et al. (2018) both detected Rhodobacterales and Alteromonadales. Additionally, studies in several reef‐building corals find both of these bacterial groups to be part of the associated microbiota (Kelly et al., 2014; Taniguchi, Yoshida, Hibino, & Eguchi, 2015). Consistent detection of these two bacterial groups in select anthozoan cnidarians may imply nonrandom associations and conserved taxa, together suggesting some biological importance. However, while differences between the animal‐associated bacteria and those in the surrounding environment suggest selection, neutral and stochastic factors may explain how these bacterial communities shift over time and between individuals (Sieber et al., 2019). For example, it is unclear whether the OTUs we identified as cycling may be a result of the anemone, interbacterial interactions independent of the host, or a combination of both factors. Future research with these OTUs to determine spatial localization and competition would be of interest.

Our comparisons of the bacterial communities associated with *N. vectensis* suggest a correlation between the presence of a circadian photoperiod and individual OTUs that exhibit a 24‐hr periodicity. To determine whether community‐level shifts are biologically important to the anemone, future research will compare individuals cultured microbe‐free to determine whether anemones have different physiology, behavior, or gene expression. At the OTU level, isolation of the identified Rhodobacterales and Alteromonadales would be useful to determine their specific impacts on host processes. This set of experiments, alongside the continued development of diverse cnidarian systems, would position this group of marine invertebrates as a comparative model for the evolution and ecology of animal–bacterial symbioses across circadian and diel photoperiods.

## AUTHOR CONTRIBUTIONS

WBL and AMR designed and conceived the study. WBL and TJC performed the experiment. WBL and TJC generated and analyzed the data. WBL, TJC, and AMR wrote and approved the manuscript.

## Data Availability

Data and supplemental files will be archived in the public archive Dryad https://doi.org/10.5061/dryad.hf33q77. Sequence data for comparative gene expression analyses are available at NCBI GEO #GSE132202.
